# Social relationships and subjective wellbeing of the older adults in India: the moderating role of gender

**DOI:** 10.1186/s12877-023-04520-x

**Published:** 2024-02-09

**Authors:** Shrestha Saha

**Affiliations:** National University of Singapore, Singapore, Singapore

**Keywords:** Intergenerational solidarity, Social participation, Multi- group analysis, Gender, Subjective wellbeing

## Abstract

**Objective:**

Social relationships have been extensively researched as having compelling health benefits. Nevertheless, these issues are understudied in low-income countries like India. This study investigates whether intergenerational solidarity or the social relationship developed in the household context and social participation or the social relationships developed in the community context are the critical determinants of subjective wellbeing (SWB) of older adults in India. In addition, this study also investigates whether gender moderates the association between social relationships and subjective wellbeing (SWB).

**Methods:**

This study utilises a latent variable approach to investigate the association between social relationships and SWB among older adults (aged 60 or older, N = 9,852) based on a cross-sectional analysis of data from Building a Knowledge Base on Population Ageing in India (BKPAI) (2011). I tested the moderating effect of gender by employing a multigroup analysis in structural equation modelling that incorporated social relationships and other predictors of SWB.

**Results:**

The study suggests that social participation has a significant protective impact on elderlies’ SWB, and gender doesn’t moderate the relation. Intergenerational solidarity has a significant health protective impact on older adults’ SWB; there is a significant moderating impact of gender. Specifically, functional and consensual solidarity have significant moderating effects of gender. Affectional solidarity, although a significant predictor of older adults’ SWB, and gender don’t moderate the relation.

**Conclusion:**

The study suggests taking into account a variety of social relationships as well as gender roles when attempting to understand SWB at a later age. The results are discussed in terms of how gender roles impact social relationships and how men and women evaluate SWB differently based on those relationships.

**Supplementary Information:**

The online version contains supplementary material available at 10.1186/s12877-023-04520-x.

## Background

Population ageing is becoming an increasingly important global issue, affecting health care and other areas of public policy in both developed and developing countries. While the natural process of ageing cannot be stopped altogether, public health policies in many developed and developing countries promote successful, healthy ageing as one of their major strategies [1, 2]. Subjective wellbeing (SWB), a key health indicator of successful ageing, is worth mentioning in this context. SWB is a reliable indicator of objective health measurements and self-evaluation of an individual’s health and life satisfaction [3]. SWB and its diverse correlates have been extensively researched in developed countries, and many studies have highlighted the importance of social relationships and social support in determining subjective wellbeing and quality of life [4, 5]. There is, however, less extensive research on these linkages in low-income settings. The impact of social relationships on older adults’ well-being in India is little studied despite having the second-largest older population in the world [6, 7]. India’s rapidly ageing population, coupled with an inadequate health system, makes it imperative to pay attention to older adults’ health and welfare needs. Specifically, this study examines how social relationships affect the SWB of older adults in India.

In recent decades, gerontological research has focused extensively on social networks and their impact on health. It has been established in a rich body of gerontological literature that individuals with satisfying social relationships have greater social support and are more socially integrated, leading to improved health and wellbeing [8–10]. Various theories across disciplines have identified the form, function, and quality of social relations and their impact on older adults’ health and well-being. Existing literature has found two primary contexts of social relations: the household context and the community /neighbourhood context. In the household context, intergenerational relationships measured by emotional bonding between parents and their grown-up children and social support exchanges among generations, such as financial assistance, tangible and practical help, and emotional support (such as expressing love and sympathy), are found to have health-promoting effects on ageing parents [11, 12]. The intergenerational solidarity framework, as proposed by Bengtson and Roberts [13], clarifies intergeneration relations as “emotional cohesion” between two generations, specially the parent-children relationship. The intergenerational solidarity model consists of six dimensions of family solidarity: affectional or emotional solidarity, associational or socially interactive solidarity, structural solidarity or locational proximity, normative solidarity or dutiful commitments, consensual solidarity or ideological similarity, and functional solidarity or instrumental social support.

In the community context, social participation or integration within individuals’ social networks has been found to benefit older adults’ physical and mental wellbeing and is generally considered an indicator of healthy ageing [8, 14, 15]. Social participation is commonly referred to as individual participation in activities (like involvement in a religious group, volunteer group, social club, etc.) that facilitates social interaction and helps to build up “meaningful social ties with others in the society or the members of social groups” [16, p. 2148]. The impact of social participation on wellbeing can be elucidated from various perspectives. Social activities influence physical and mental wellbeing by lessening the risk of social isolation, encouraging social proximity, providing emotional support, reinforcing social roles, and providing a sense of being esteemed [4, 17]. Social participation enhances social support, which helps to combat psychological distress and depression [18, 19].

While the health-protective impacts of social relationships are common to all societies, types of social relations are largely influenced by cultural values and practices shared across cultures. Prior gerontological literature has frequently applied a culturally relevant theoretical framework to analyse social relationships and health. Consequently, research on family and community-based social relationships is country-specific and primarily divided into two prototype cultures: individualism and collectivism. There has been a growing interest in intergenerational solidarity theory in Asian countries with a collectivist social ethos, high intergenerational ties and filial piety, and a lack of formal social support for older adults. Significant implementation of intergenerational solidarity theory in ageing research has been found in Asian countries like China [20, 21], Thailand, Vietnam, Myanmar [22], and India [23, 24]. In contrast, social participation or community-based social relations as an indicator of health has gained much validation in Western cultures stemming from the individualistic ethos that promotes personal independence. Much research in Western nations has endorsed that many elder parents become increasingly dependent on their communities for social support, companionship, and recreation as they age [25, 26].

However, this study argues that family-based relationships in the household context and social relationships in the community context constitute two distinct types of social ties; the former involves a more intimate relationship, while the latter involves greater social interaction outside the familial context. Thus, their role or relative contributions to health protection may differ. This study extends the existing literature by defining older adults’ social networks as family and community-based relationships to provide a more comprehensive picture of social relationships in the context of India.

Although social relationships are valuable resources across various social contexts, they are not equally accessible to everyone [27]. Research in both collectivistic and individualistic cultures has demonstrated considerable gender differences in qualitative and quantitative aspects of social relations, and gender frequently moderates the impact of social relationships on health and wellbeing [28, 29]. Such differences underscore the need to explore the linkages between social relationships and wellbeing in later life through the perspective of gender. Thus, in addition to the types of social relationships and their impact on SWB, a related issue is the extent to which social relationships amplify gender inequality in SWB.

The present Study In India, the intergenerational residence has been seen as a collaborative social network which supports older adults, most notably for their health and wellbeing, more than in the West due to cultural values and economic necessities [23, 30, 31]. However, the rapid demographic changes (like the decline in fertility rate and expanded longevity) along with the drastic transformation of socio-cultural (small family norms, increasing divorce rate) and changes in family values (like attitudinal changes towards filial piety) have weakened the traditional living arrangement of the older adults in India [32, 33]. In recent years, there has been a significant transformation in the structure and living arrangements of Indian families, particularly concerning older adults. The traditional multigenerational households, known for their focus on collective well-being and deep respect for elders, are gradually transitioning into nuclear households. These nuclear families are characterised by values of independence, individuality, and privacy [34, 35]. As the country ages and its intergenerational residence rates decline, fewer opportunities exist for elders to receive instrumental and emotional support from adult children, resulting in tremendous health care and economic challenges for older adults. Due to these facts, it seems relevant to investigate whether social relationships developed outside the family context are increasingly influencing the health and wellbeing of older Indians. Recently, the importance of social participation for older adults has been emphasised in policy discussions due to its benefits for enhancing physical and mental health and bolstering resilience against stress, depression, and loneliness [17, 36]. However, less attention is given to the impact of social participation on older adults’ health in the Indian context. This paper argues that social participation deserves more attention as a factor in the health of older adults in India, especially in light of the country’s significant social and institutional changes that have weakened intergenerational ties and family support exchanges between generations.

Furthermore, the complexity of intergenerational relationships and how they affect Indian elders’ wellbeing has not been adequately explored by research in family demography and gerontology. The aspect of family relations in Indian gerontological literature has been typically conceptualised and measured through the structural component of a household, i.e. the composition of household members [23, 24]. While the living arrangement is one aspect of family relationships and intergenerational solidarity, much remains to be explored about family dynamics and the wellbeing of older adults. The current study is inspired by the intergenerational solidarity theory proposed by [13], which assesses multi-dimensional aspects of intergenerational relationships and their impact on older adults’ subjective wellbeing. The ‘intergenerational solidarity’ theory proposed by Bengtson and Roberts [13] provides a pertinent theoretical backdrop for investigating the relationships between two successive generations and how they relate to health in later life. The theory clarifies intergenerational relations as emotional cohesion between two generations specially, especially between parents and their children. Central to this framework is the concept of mutual support and reciprocity across generations. Bengtson and Roberts [13] laid out this theory, outlining six core dimensions of family solidarity: affectional solidarity (emotional ties), associational solidarity (social interactions), structural solidarity (geographical closeness), normative solidarity (sense of duty), consensual solidarity (ideological alignment), and functional solidarity (practical support).

The current study also assumes that the pattern and impact of intergenerational solidarity and social participation on, older adults’ subjective wellbeing vary by gender. Gender-specific social roles and norms influence the structure and composition of a person’s social network across a wide range of social contexts and the kinds of resources they have access to [27, 37]. In the household context, the intergenerational relationship is a complex process involving multifaceted support exchanges among family members and governed by gender values and norms across generations. In both collectivist and individualistic societies, gender-specific socialisation has conditioned women to prioritise family ties more emotionally and to be more responsible for domestic work and daily care while adult male members provide financial assistance [38–40].

Additionally, women and men were shown to have distinct needs for social attachment and expectations from network relationships, and there is plenty of research to suggest that they share different social activities and wellbeing relationships [41, 42]. Research on both collectivist and individualistic societies has also demonstrated considerable gender differences in diversity, frequency of social participation and the composition of social ties, and their impact on health outcomes [40, 43, 44]. Given gendered societal norms, it is crucial to investigate whether the pattern of social relations and their impact on health varies across gender.

## Research framework

The current study uses a latent variable approach to examine the relationship between social relationships and subjective well-being (SWB). Instead of using aggregated or composite scores, this approach provides a nuanced understanding by treating complex constructs as latent variables derived from multiple observed indicators. Using a latent variable approach is becoming increasingly popular in modern family dynamics research due to its effectiveness in examining familial bonds and intergenerational support exchanges [45–47]. Techniques used include Confirmatory Factor Analysis (CFA) for its strength in theory-driven relationship assessment and Structural Equation Modelling (SEM) to assess causality.

**Fig. 1:**
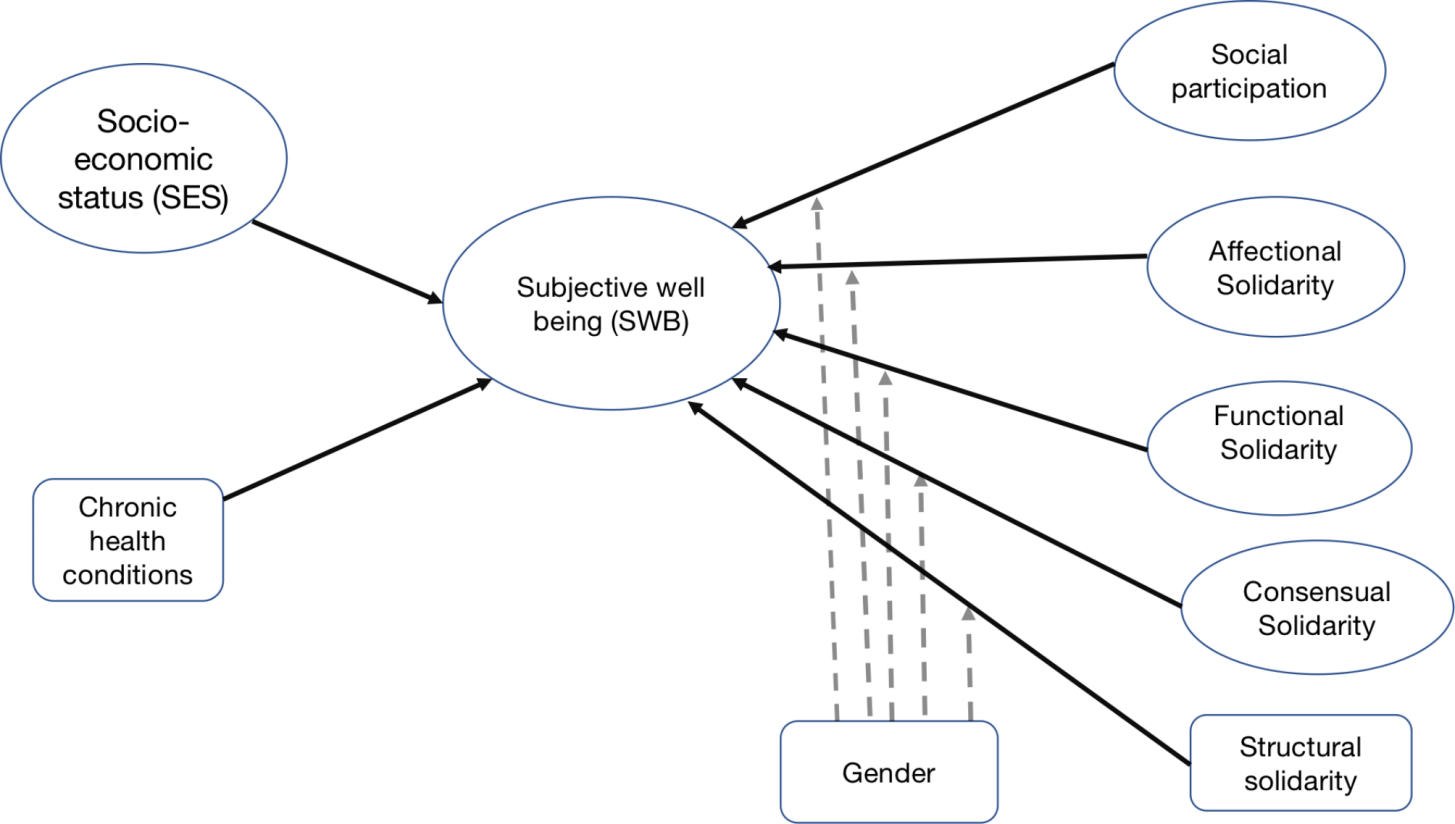
Conceptual framework: Solid arrows indicate predicted causal relationships among social relationships and Subjective Wellbeing, and dashed arrows indicate the potential moderating effects of gender on each causal linkage examined whether the relationship between the independent and dependent variables varies across gender

The presented conceptual model (Fig. 1) elucidates the interconnections between various dimensions of intergenerational solidarity and the role of social participation. Specifically, it aims to highlight the effects of social relationships within the household and the broader community on the subjective well-being (SWB) of older adults in India. Additionally, this model theorises that gender might serve as a potential moderator in these relationships. The structure of the model is theory-driven; it aligns with the multi-dimensional framework of ‘intergenerational family solidarity’ posited by Vern Bengtson and colleagues, as observed in U.S. data [13]. While their model outlines six dimensions of solidarity—structural, associational, consensual, affectional, functional, and normative, however due to the constraints of available data, this analysis narrows its focus to four core dimensions. Social participation is examined through a singular, overarching facet in this study.

### Data

This study utilises cross-sectional data - Building a Knowledge Base on Population Ageing in India (BKPAI) (2011) in India. Data collection has been approved by the Ethics Committee at Southampton University. All participants provided informed written consent, and data were kept confidential and used only for research purposes. This manuscript was based on informed consent obtained from all literate participants and their legal guardians. The survey has collected data on socio- demographic characteristics, family relations, health status, and old age security from seven states of India, namely, Kerala, TamilNadu, Maharashtra, Himachal Pradesh, Punjab, Odisha and West Bengal, which have a higher proportion of older adults (above 60 years) population and from the household have at least one older adult. The total sample size is 9852.

### Dependent variables

***Subjective Wellbeing***: SWB is measured by nine items capturing an evaluation of one’s life in terms of overall life satisfaction. The answer is taken by a three-point psychometric scale (1,2,3), with 1 representing a poor and three good evaluations of life satisfaction. Taking nine answers for 9 items (with an α coefficient of 0.88), the total score of SWB ranges from 9 to 27, where a higher value represents the better evaluation of life satisfaction. Respondents were asked: if they think life is interesting, feeling about present life, level of happiness, feeling about social status and standard of living, feeling about life achievement and success, feeling about accomplishment, control on life circumstances, confidence to manage a difficult situation in the near future, and confidence in coping with the future.

### Independent variables

***Social participation***: Social participation is measured by how often the older adults have engaged in three distinct social activities over the past 12 months: attending any public meeting, attending any group, club, or organisational meeting, and working with other people in your neighbourhood in the past 12 months. The frequency of social participation is categorised and coded into three broad groups 0 = never, 1 = occasionally, 2 = frequently. Social participation consisted of three items with an α coefficient of 0.80.

### Intergenerational solidarity

*Structural solidarity*: This study explores structural solidarity within the framework of Bengtson and Roberts’ theory [13], which posits that the residential propinquity of family members provides an ‘opportunity structure’ that can facilitate intergenerational interactions and support systems. In their study, Bengtson and Roberts [13, p.862] utilise residential proximity between parents and children as a tangible measure of structural solidarity. Aligning with their theory, this paper sheds light on the geographical proximity of two generations, specifically, whether aging parents reside with their adult children (male) as a proxy indicator of Structural solidarity. The study adopts a dichotomous approach to categorise older adults’ living arrangements: either living alone/with others or living with an adult son. While Bengtson and Roberts did not explicitly dichotomise living arrangements, their emphasis on residential propinquity (measured by residential proximity between parent-child) provides the theoretical grounding for such an approach.

The simplified binary categorisation of older adults’ living arrangements are employed to convey both the geographical closeness of ageing parents to their children and the representation of Indian cultural values, where living with an adult son signifies strong intergenerational connections [20, 23, 24]. The dichotomisation of the variable serves the purpose of maintaining a parsimonious approach to the analysis. Accordingly, the “living with an adult son” category is thus considered the closest form of residential proximity due to minimised geographical distance and conducive to more intergenerational interaction, whereas “living alone or with others” indicates lesser closeness.

The original questionnaire used for the study provides five distinct living arrangement categories: living alone, living alone with a servant, living with a spouse only, living with a spouse and servant, and all others. Additionally, the questionnaire provided information on (1) whether aging parents reside with their children vs. all others and (2) the age and gender composition of the residing children. Considering these variables, this study streamlined the categories into two primary groups: Structural solidarity = 0 when aging parents not living with an adult son, and Structural solidarity = 1, when aging parents are living with an adult son.

#### Functional solidarity

Prior research in intergenerational relationships and family sociology has frequently used the concept of functional solidarity, encompassing various forms of support, both tangible and intangible, to understand family dynamics more comprehensively. Tangible support incorporates practical help, fixing something around the house, grandparenting and household chores. Intangible support includes advising on important matters about things they could do or help with a decision; emotional support like listening to concerns or being available when the other is upset [48–50]. Considering the previous literature, this study incorporates four variables. Functional solidarity for this study is measured by the support provided by older adults in various household tasks in the last 12 months like Grandparenting, Handling household chores, providing advice to the children and resolving a dispute. The answer was taken dichotomously and coded as no = 0 yes = 1. The α coefficient of these four items is 0.65. In the given dataset, the responses are provided by theolder adults, reflecting their self-perceived roles and contributions within the household.

#### Affectional solidarity

Affectional solidarity is measured by the perceived emotional attachment of older adults to their families. Respondents were asked: if they think they are essential to their family and if they think that their family members think of them as important in their family. The answer is taken on 3 points psychometric scale (not important = 0, somewhat important and very important = 2). The α coefficient of these two items is 0.87.

#### Consensual solidarity

‘Consensual Solidarity’ in this study pertains to the measure of ideological alignment between older parents and their children. The Central assumption is that when strong intergenerational ideological similarities exist, decision-making becomes a collective effort involving multiple members, including older adults, and results in a cooperative approach to household choices. The questions were asked to the older adults on their role in household decision-making for significant events like the wedding of a son/daughter, property purchases and sales, purchasing household items, giving of gifts to relatives, children’s and grandchildren’s education, and organising social events. The responses captured as No role = 1 The older adults do not participate in significant household decisions. Alone = 2 Decisions are made solely by the older adults. With spouse = 3 The older adults collaborate with their spouse to make decisions. With children = 4 The older adults and their children jointly make decisions. With everyone = 5, The older adults are involved in a collective decision-making process that includes all adult household members. The α coefficient of these six items is 0.88.

### Other covariates

The other predictor includes individual socioeconomic characteristics (measured by education and household income) and health status (measured by the number of chronic health conditions).

## Methods

The background characteristics of the respondents, as well as the prevalence of SWB are analysed using univariate and bivariate analyses. Pair-wise correlations are performed between the variables to ascertain whether there is a meaningful association between SWB and the other variables. A measure of internal consistency among items was determined by using Cronbach’s alpha for latent variables that consist of multiple observed items.

I use latent variable Structural equation modelling (SEM) to test the hypothesised model (Fig. 1). SEM is a more robust alternative to conventional approaches like regression when testing hypothesised relationships between latent constructs due to its ability to simultaneously handle measurement error and structural components. As the hypothesised model incorporates a moderating variable (gender), I conducted a multigroup analysis in SEM to determine gender groups. With this multigroup analysis, measurement and structural invariance are tested directly across gender groups (moderating variable), thus ensuring that observed differences in structural relationships among gender groups are not caused by measurement errors or measurement differences [51].

The analysis consists of two parts; in the first part, I evaluate confirmatory factor analysis (CFA), followed by a structural model for the entire sample. The first step is to identify the structural relationships among the variables. The measurement model describes the relationships between scale items and their underlying factors (latent constructs), while the structural model describes how those constructs are causally connected. The structural model offers the characteristics of general regression analysis and the possibility of simultaneously estimating correlations.

The second part of the analysis involves assessing the moderating effect of gender using a multigroup comparison. As a prerequisite of multigroup comparison, Measurement Invariance (MI) has been tested to see whether the measurement parameters (e.g., factor loadings) are operating consistently for both gender groups. This step is required before analysing the structural model as it ensures that differences across groups do not stem from measurement differences but from structural differences. MI is accomplished by comparing several models, starting with an unconstrained baseline model, known as a configural model, which imposes no equality constraints across groups, and comparing it to its next-level model with greater constraints. Once measurement invariance is established, moderating effect of gender is tested through a multigroup approach. In multigroup SEM, moderating effects can be tested by forcing structural path coefficients to be equal across groups (i.e. moderating variables, gender). If the constrained model (path coefficients forced to be equal across groups) significantly differs from the unconstrained model, then a moderating effect exists.

To evaluate whether the model fits the data, the study report Chi-square statistic and other fit indices like the comparative fit index (CFI), Root mean squared error of approximation (RMSEA), and Standardized root mean square (SRMR) to evaluate the model fit. Although chi-square is the traditional and most commonly used measure for assessing the model fit [52], its sensitivity to sample size cannot be ignored. CFI ≥ 0.90. and RMSEA and SRMR values < 0.08 suggest acceptable model fit [51].

**Table 1:** Descriptive characteristics of the variables

Variables	Description		mean/ percentage	SD	N	range /categories
Health Indicators						
Subjective Wellbeing (SWB)	Subjective well being		17.11	3.94	9,574	9–27
Chronic health conditions	number of chronic diseases		1.20	0.89	9852	0–6
Socio economic status (SES) and demographics	Age		68.00	7.20	9,852	60–120
	Gender	male	47.42		4,672	0 = male 1 = female
		female	52.58		5180	
	Education	no formal schooling	46.27		4,533	
		5 years of schooling	20.37		1,996	
		6–10 years of schooling	24.87		2,437	
		11 years and more	8.48		831	
	Household wealth quintile	low income	39.89		3,928	0 = low income 1 = high income
		high income	60.11		5,918	
social participation			0.99	1.50		0–6
Intergenerational solidarity	Affectional		4.53	1.41	9,823	0–6
	Structural	Not living with an adult son	42.43		4,180	0 = Not living with an adult son 1 = living with adult son
		Living with adult son	57.57		5,671	
	Functional		4.19	1.89	9839	0–7
	Consensual		18.70	7.22	9,852	0–30

Source: BKPAI (2011)

## Analysis and results

### Descriptive statistics and bi-variate analysis

Table 1 shows that the overall sample shows the mean score of SWB is 17. SWB is measured on a scale that ranges from 9 to 27, with higher scores indicating a higher level of subjective well-being and lower scores indicating a lower level. So, the average score of 17 suggests that, on average, the older adults individuals in the sample reported a moderate level of well-being. The mean number of chronic diseases is 1.20 on average, individuals in the sample tend to have one or more chronic diseases. The gender composition of the sample size shows a higher proportion of females, 52%. The pairwise correlation (Table not shown) shows that the majority of these variables are correlated within a moderate range, suggesting that multicollinearity would not pose a significant problem. SWB positively correlates with family solidarity and social participation. The SWB of females is likely to be poorer than the SWB of males.

### Structural equation models

The CFA assesses the measurement properties of six latent constructs subjective wellbeing, affectional solidarity, functional solidarity, consensual solidarity, social participation and socioeconomic status. As shown in Table 2, the measurement model provides a good fit of the theoretical model to the given data in all fit indices except for the chi-square statistic, as it is sensitive to the sample size. The CFI = 0.93, RMSEA = 0.05 and SRMR = 0.04. Moreover, the unstandardised factor loadings in Table 2 indicate how strongly a scale item is associated with its latent factor. The item with highest factor loading is fixed at 1to provide a clear reference point for comparing the loadings of other items.

After achieving the validity of the measurement model, I test the hypothesised structural model, i.e. the effect of all major predictors and control variables with the outcome variable for the full sample (Table 3). The goodness of fit indices in Table 3 shows an acceptable fit (CFI = 0.91, RMSEA = 0.05, SRMR = 0.05) of the hypothesised model to the given data; hence, it demonstrates empirical support of the hypothesised relationships.

#### Moderating role of gender

I assessed measurement invariance across gender groups through multigroup CFA, using chi-square difference tests for a set of nested models. According to [53], multigroup analysis of measurement invariance begins with the establishment of a baseline (configural) model in which model parameters for each group are estimated separately, and no equality constraints are imposed across groups. By fitting this configural model, a baseline χ2 value is generated against which a range of increasingly restrictive models of invariance can be compared to identify the source of non-invariance. As the Chi-square difference test is sensitive to sample size, differences in Comparative Fit Indices (CFI) are used to define invariance [54]. A change in CFI lower than or equal to -0.01 indicates a similar fit between the two models. Table 4 summarizes the baseline model fit to the configural invariance held, suggesting that both groups were adequately represented by the factor structure (CFI = 0.93, RMSEA = 0.051). Similarly, the model fit indices for the weak, strong, and strict invariance models indicated adequate model fit.

**Table 2:** Results of confirmatory factor analysis (N = 9506)

Subjective wellbeing	β coefficients(Unstandardised estimates)
Compared with the past, do you feel your present life is very happy/ quite happy/ not so happy? (SUBI 1)	1
Do you feel your life is interesting? (SUBI 2)	0.893***
On the whole, how happy are you with the kind of things you have been doing in recent years? (SUBI 3)	0.994***
Do you think you have achieved the standard of living and the Social status that you had expected? (SUBI 4)	0.915***
How do you feel about the extent to which you have achieved success and are getting ahead? (SUBI 5)	0.932***
Do you normally accomplish what you want to? (SUBI 6)	0.902***
Do you feel you can manage situations even when they do not turn out as expected? (SUBI 7)	0.934***
Do you feel confident that in case of a crisis (anything that substantially upsets your life situation) you will be able to cope with it/face it boldly?(SUBI 8)	0.941***
The way things are going now, do you feel confident in coping with the future? (SUBI 9)	0.979***
**Affectional solidarity**	
To what extent you think you are important to your Family (AFF 1)	1
To what extent you think that your family members feel about your importance to the family (AFF 2)	0.93***
**Functional solidarity**	
Settling dispute (FNC 1)	1
Taking care of household chores (FNC 2)	0.592***
Giving advice to the children (FNC 3)	0.872***
Taking care of grandchildren (FNC 4)	0.426***
**Consensual solidarity**	
Property purchases and sales (CNS 1)	1
Children’s and grandchildren’s education(CNS 2)	0.949***
The giving of gifts to relatives.(CNS 3)	0.969***
Wedding of a son/daughter (CNS 4)	0.902***
Purchasing household items.(CNS 5)	0.99***
Arrangement of social and religious events (CNS 6)	0.829***
**Social participation**	
How often in the last 12 months have you attended any public meeting in which there was discussion of local, community or political affairs? (SP 1)	1
How often in the last 12 months have you attended any group, club, society, union or organizational meeting? (SP 2)	0.954***
How often in the last 12 months have you worked with other people in your neighborhood to fix or improve something?(SP 3)	0.863***
**Socio economic status (SES)**	
Education level (SES 1)	1
Income (SES 2)	0.957***

CFI = 0.929, RMSEA = 0.052, SRMR = 0.03 *p < 0.1,**p < 0.05,***p < 0.01

**Fig. 2:**
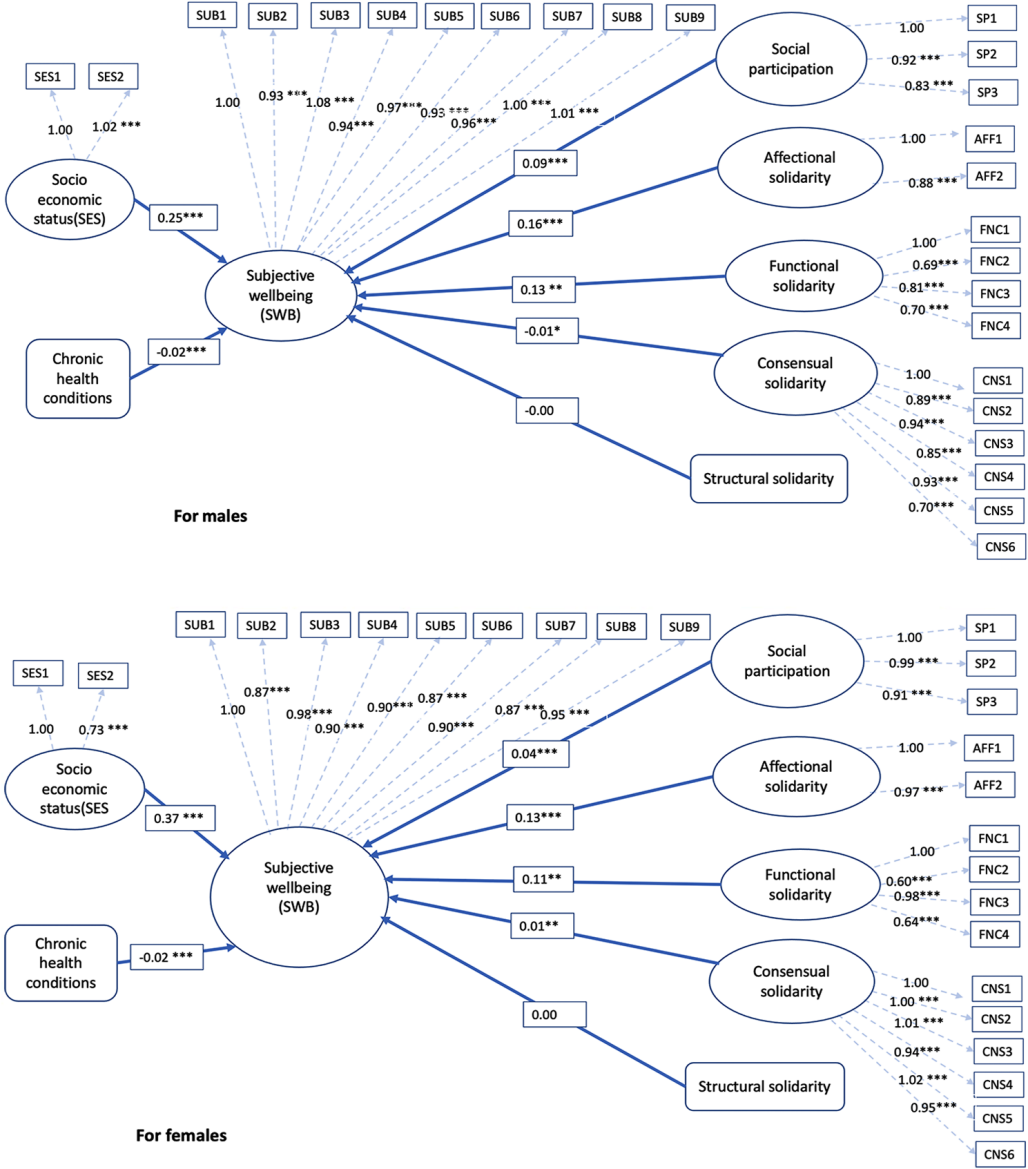
Solid arrow indicates path coefficients (unstandardised coefficient) of structural models for males and females. The dashed arrow indicates factor loadings of observed variables with their underlying latent construct. Notes: ∗*p* < 0.05 ∗∗*p* < 0.01 ∗∗∗*p* < 0.001

Once the measurement model was confirmed to be group-invariant, the next step was determining if gender moderated the model. The first step is constructing a baseline unconstrained model for the gender groups using a nested multigroup approach. The resulting model (Table 5) has an acceptable fit (χ2 = 9880, df = 668, *p* < 0.001, CFI = 0.91, RMSEA = 0.05, SRMR = 0.04). Figure 2 indicates that only structural solidarity does not have any significant impact SWB, for men and women. For women, affectional, functional and consensual solidarity, as well as social participation, have a significant positive impact on SWB.

Further, all path coefficients are constrained to be equal across gender groups to achieve a fully constrained model. The fully constrained model gives an acceptable fit (χ2 = 9913, df = 675, *p* < 0.001, CFI = 0.91, RMSEA = 0.05, SRMR = 0.05) and produces significant increase in chi-square and degrees of freedom. The model comparison by chi-square difference test between the unconstrained and the fully constrained model reveals a significant difference (∆ χ2 = 33 ∆ = 7, *p* < 0.001) of structural coefficients across groups, thereby confirming the moderating role of gender (Table 6).

To acquire more persuasive evidence of non-invariance in path coefficients between the two gender groups, the study repeated the SEM multigroup analysis followed by model comparisons using a series of chi-square difference tests. Comparisons were then made between the fully constrained model and a set of less constrained models. In each less constrained model, one path coefficient is estimated without constraint (i.e. estimated as a free parameter) to verify its non-invariance (Table 6). Chi-square difference tests are performed to identify which paths are significantly different. This procedure more explicitly tests the moderating effect of gender on each path coefficient separately. Through the iterative model comparison process, it has been found that the paths from functional solidarity and consensual solidarity differ significantly in each group. Overall our data appeared to support the existence of moderation of gender (Table 6).

**Table 3:** Path coefficient from the structural model

Subjective wellbeing	$\beta$ (Unstandardized estimates)
Social participation	0.56***
Structural solidarity	0.003
Affectional solidarity	0.133***
Functional solidarity	0.123***
Consensual solidarity	0.003
SES	0.29***
Chronic Health	-0.023***

*p < 0.1,**p < 0.05,***p < 0.01. CFI = 0.912, RMSEA = 0.054, SRMR = 0.047

**Table 4:** Measurement invariance across gender groups(N = 9506)

	CFI	diff. CFI	RMSEA
Configural invariance (Baseline model)	0.92	NA	0.052
Weak invariance (factor loading invariant)	0.92	0	0.051
Strong invariance (factor loading and intercept invariant)	0.91	0.01	0.054
Strict invariance (factor loading and intercept and mean invariant)	0.90	0.02	0.057

**Table 5:** Path coefficient of Multigroup SEM (unconstrained)

Subjective wellbeing	Male (N = 4499) $\beta$ (unstandardized estimates)	female (N = 5007) $\beta$ (unstandardized estimates)
Social participation	0.091***	0.047***
Structural solidarity	-0.004	0.002
Affectional solidarity	0.156***	0.135***
Functional solidarity	0.131**	0.117**
Consensual solidarity	-0.013*	0.012**
SES	0.258***	0.374***
Chronic Health	-0.022***	-0.027

CFI = 0.909, RMSEA = 0.054, SRMR = 0.048 *p* < 0.1,**p < 0.05,***p < 0.01

## Discussion

Based on a sample of older Indians (age 60 and above), this study identified patterns of intergenerational solidarity and social participation and then assessed their effects on subjective wellbeing. Additionally, it delved deeper into gender differences, recognising that men and women may have different social relationship patterns and that the impact of social relationships on SWB may differ between them. The result from the full sample shows that social participation is a significant predictor of SWB. Such evidence is crucial in India, where family interdependence and family-based social relations are often emphasised for the well-being of older adults, while social ties outside the family are not emphasised much as potentially contributing factors to health. In the Indian cultural context, older adults’ health and well-being are often discussed concerning strong intergenerational ties, and norms of filial piety [23, 30]. However, traditional family based care and intergenerational family norms are gradually declining in India due to demographic changes and corresponding changes in social economics and ideologies [30, 33]. Such changes in demographic structure, social norms, and economic conditions may explain why older adults engage more in social activities to enhance their subjective wellbeing.

The result from the unconstrained model (Table 5) also reveals that the family-based social relation measured by affectional solidarity and functional solidarity strongly predicts a higher level of SWB for both males and females, although the magnitude varies across genders. Affectional solidarity, as defined by the closeness to one another, is a predictor of wellbeing and life satisfaction among older adults, particularly in East Asian cultures, where filial piety plays a crucial role in the parent-child relationship [55, 56]. The emotional bonding helped to maintain connections between generations and gave children a sense of duty to support their parents.

In addition to affectionate solidarity, older parents providing help and support in different spheres of daily life, such as personal care, household chores, financial assistance, and grandparenting, significantly affect their SWB. Several studies in different social contexts report similar observations regarding functional solidarity and the wellbeing of the older adults. According to previous research, older parents maintain more positive psychological states when they can reciprocate in their families through financial support or other instrumental assistance [55, 56].

Consensual solidarity has shown a significant negative impact on older men’s SWB, whereas it has a significantly positive effect on older women’s SWB. This means having joint or egalitarian decision-making at the household level have a negative impact on the SWB of older adults, but for women, having more people make joint decision shows health a protective impact. Differences in the influence of consensual solidarity on older adults’ subjective well-being (SWB) can be rooted in societal and household dynamics. Historically, as men have been positioned as primary decision-makers, shifts towards egalitarian practices might cause feelings of lost control, negatively influencing their SWB (Courtenay, 2011). Conversely, older women could see collaborative decision-making as a source of empowerment, enhancing their autonomy and overall well-being [57, 58]. Further, this study does not find any significant relation of structural solidarity measured by living in the same household with an adult son compared to not living with an adult son (living alone and with others). This insignificant coefficient (β = 0.00), is unexpected as living with an adult son and his family is the most appropriate living arrangement for older parents to maintain filial piety in Asian societies including India. It is generally presumed that co-residential living arrangements, denoting physical closeness, would facilitate intergenerational support and thereby enhance well-being. This finding challenges the traditional belief that co-residing with male offspring inherently improves well-being. Despite cultural esteem for this living arrangement, contemporary factors such as socio-economic shifts, urbanisation, and changing family dynamics may reshape this tradition [30, 33]. Additionally, concerns about older adult abuse within extended family setups in India raise the possibility of negative effects on the health and well-being of older adults [59, 60]. The inconclusive impact of structural solidarity on SWB highlights the need for a more intricate evaluation of living arrangements vis a vis structural solidarity, moving beyond the traditional binary to include diverse living scenarios that might capture the contemporary intergenerational dynamics.

A comparison between unconstrained and constrained models shows the moderating role of gender on the association between social relationships and health (Table 6). Gender does not moderate the relationship between social participation and SWB; nevertheless, intergenerational solidarity measured by consensual and functional solidarity acted in significantly different ways for older men and women.

**Table 6:** Multigroup analysis: Testing for path coefficients invariance across gender groups (N = 9506)

	χ^2^	df	Δχ^2^	Δdf	Pr(> Chisq)	Moderating effect of gender
Model 1: Baseline model (factor loading invariant)	9880.8	668				
Model 2: Factor loadings and all path coefficients invariant (fully constrained)	9913.8	675	33.02	7	0.000	Yes
Model 3 Path coefficient (unconstrained) social part–>SWB	9912	674	1.51	1	0.22	No
Model 4 Path coefficient (unconstrained) structural–>SWB	9912	674	1.98	1	0.16	No
Model 5 Path coefficient (unconstrained) Affectional–>SWB	9913	674	0.79	1	0.37	No
Model 6 Path coefficient (unconstrained) Functional–>SWB	9910	674	3.59	1	0.05	Yes
Model 7 Path coefficient (unconstrained) consensual–>SWB	9904	674	9.67	1	0.001	Yes

The findings indicate that consensual solidarity measured by egalitarian decision-making or joint decision-making practices at the household level has a negative impact on subjective wellbeing for the male older adults. However, for females, consensual solidarity has a positive impact on SWB. This difference highlights the significant moderating effect of gender. This may be because older men, who are more likely to be raised according to patriarchal gender norms, feel more empowered when they hold the authority of household decision-making alone or with their spouses. Studies across different social contexts have found that individuals’ autonomy in decision-making enhances their sense of personal mastery and psychological resilience [61, 62]. Research has shown that even physically weak men retained their self-image as the “head of the household,” keeping control over intrahousehold decision-making [63]. Such a finding suggests that in India, where older men typically exercise inegalitarian headship, it gives them a sense of stability, control over resources, and the power to govern their life conditions.

Further, functional solidarity (measured by the instrumental support provided by older adults in various household tasks), although it has a positive influence on SWB for men and women, the magnitude of the relation is significantly moderated by gender. Indian families are still headed mainly by men, and instrumental support exchanges within families revolve around patriarchal values [64]. Despite mounting evidence of sex role transformation, older women both in developed and developing countries continue to be more involved in household chores, while older males often spend time outside the home and have only slightly expanded their contributions to domestic work [65, 66]. Numerous studies suggest that although men and women perform distinct activities to ensure the allocation of goods and services for domestic needs, women’s wellbeing is adversely affected by the considerable burden of unpaid domestic work and informal caregiving. Additionally, they sacrifice their social life while performing the gendered tasks, endure more stress and pressure than men, and rarely ask for outside help when necessary [67, 68].

## Policy implications

This study has significant ramifications for future research and strategies that aim to improve public health through social relations. A key finding of the study is the culture of filial piety, and family bonds are still highly valued, so policies supporting co-residence with children can positively affect older adults’ subjective well-being. Since India lacks an old age security system and social pension scheme to support older adults, the bulk of people in old age become dependent on young family members for economic security and long-term health care, making family the greatest option for older adults care [69, 70]. Financial assistance from immediate family members, especially children, is an adequate replacement for the older adults’ insufficient personal wealth and the lack of assistance from the government. The policy should uphold the traditional values of filial obligations and encourage collaboration among stakeholders in the Indian context.

Intergenerational relations are associated with SWB in a different way for males and females. Understanding and addressing gender patterns of social relations is important since some forms of gender norms, like involvement in kin keeping and higher responsibility for household management, can foster and support lifetime social bonds. However, other forms of gender norms and restrictions, like too much involvement in caregiving may increase social isolation, especially in older women. Thus from a public health policy perspective, this study calls for further research to examine gender differences in intergenerational relations and tailor social policies to encourage gender equality.

Social participation has a considerable positive effect on SWB among older adults regardless of gender. In India, where intra-familial insurance and support are traditionally regarded as health benefits, this finding could offer policymakers promising avenues to implement pertinent policies that motivate senior citizens to participate in various formal and informal social activities. In particular, it would benefit those older adults with weak or absent informal ties with family, relatives and kin.

### Limitations and future research

Given the limitations of this study, the findings call for additional research. Although the study empirically confirms the positive impact of social relationships at both the household and contextual levels on older adults’ SWB, the study does not provide any insights into the pathways that lead to these effects. For instance, several research in recent years has investigated how different support exchanges across generations boost personal mastery and develop a sense of self-esteem, which in turn increases the degree of social support, reduces low feelings and increases life satisfaction [40, 47]. To better comprehend the intricate connections between intergenerational relationships and SWB, an integrative framework is required to delineate the pathways between these two phenomena.

The study emphasises the perspectives of older adults on their roles and contributions in the household without offering a comparative view from their children or other household members. This narrow focus might lead to a skewed understanding of intergenerational solidarity. For example, when considering household decision-making or conflict resolution roles, the older adults might either diminish or exaggerate their contributions. Gaining insights from younger family members could have presented a more diverse, or even opposing, viewpoint. The absence of this multi-generational lens is a clear limitation in our current analysis.

While this study recognises the frequency of social participation in the community as a determinant of SWB, the qualitative aspects of social participation, such as the role in social activities, the length of activities, and the quality of social relations, were not assessed in the survey and could not be taken into account in the analysis. It is critical to identify explicitly the underlying mechanisms and qualitative features of social relationships that produce these beneficial effects.

### Conclusion

In line with earlier research from India and elsewhere, this study concludes that families are an essential source of interpersonal interaction, mutual support, and pooled resources that provide older parents with informal protection in times of need. The study also highlights that maintaining relationships with people outside one’s immediate family is crucial as one ages. The function of social participation as a protective element in India’s familial culture is supported by limited empirical evidence, as was mentioned in the introduction. In light of these findings, future research should examine whether different types of informal support are related to social isolation, wellbeing, and social inequalities among older adults. It is essential to put active and healthy ageing at the forefront of policy agendas so that people can enjoy better health and more autonomy and engagement with their surroundings as they age. Finally, social relationships are undoubtedly valuable resources, but as the study demonstrates, not everyone has access to them. This calls for concentrating governmental efforts and resources on identifying people who are frequently at risk (for instance, depending on gender or race) and giving them a chance to build and nurture social relationships from early childhood until the end of life.

### Electronic supplementary material

Below is the link to the electronic supplementary material.

Supplementary Material 1

## Data Availability

The study utilizes a secondary data which is available on request from director@isec.ac.in at http://www.isec.ac.in/.
