# Supplementary material for: Social relationships and subjective wellbeing of the older adults in India: the moderating role of gender
Source: BMC Geriatr. 2024 Feb 9;24:142. doi: 10.1186/s12877-023-04520-x (PMC10854134; doi:10.1186/s12877-023-04520-x)
Supplement: Supplementary file 1 — Supplementary Material 1 [file 12877_2023_4520_MOESM1_ESM.docx]

|  | Female | SWB | Chronic diseases | Affectional | Social part | Functional | Consensual | Structural | Wealth  Quintile | Education |
| --- | --- | --- | --- | --- | --- | --- | --- | --- | --- | --- |
|  |  |  |  |  |  |  |  |  |  |  |
| Female | 1 |  |  |  |  |  |  |  |  |  |
| SWB | -0.11* | 1 |  |  |  |  |  |  |  |  |
| Chronic diseases | 0.06* | -0.04* | 1 |  |  |  |  |  |  |  |
| Affectional | -0.15* | 0.33* | -0.01 | 1 |  |  |  |  |  |  |
| Social part | -0.22* | 0.17* | 0.02* | 0.08* | 1 |  |  |  |  |  |
| Functional | -0.09* | 0.27* | -0.07* | 0.39* | 0.20* | 1 |  |  |  |  |
| Consensual | -0.09* | 0.15* | 0.02* | 0.25* | 0.15* | 0.28* | 1 |  |  |  |
| Structural | 0.03* | 0.03* | 0.03* | 0.00 | 0.03* | 0.02 | 0.22* | 1 |  |  |
| Wealth_quint | -0.04* | 0.32* | 0.12* | 0.17* | 0.10* | 0.12* | 0.15* | 0.14* | 1 |  |
| Education | -0.32* | 0.33* | -0.01 | 0.25* | 0.13* | 0.20* | 0.05* | -0.06* | 0.37* | 1 |

Supplementary Table 1: Correlation among variables used
